# Long-Term Effects of Induced Hypothermia on Local and Systemic Inflammation - Results from a Porcine Long-Term Trauma Model

**DOI:** 10.1371/journal.pone.0154788

**Published:** 2016-05-04

**Authors:** K. Horst, D. Eschbach, R. Pfeifer, B. Relja, M. Sassen, T. Steinfeldt, H. Wulf, N. Vogt, M. Frink, S. Ruchholtz, H. C. Pape, F. Hildebrand

**Affiliations:** 1 Department of Orthopedic Trauma and Harald Tscherne Research Laboratory, University Hospital Aachen, Pauwelsstraße 30, 52074 Aachen, Germany; 2 Department of Hand, Traumatology and Reconstructive Surgery, University Hospital Marburg, Marburg, Germany; 3 Department of Trauma, Hand and Reconstructive Surgery, Goethe University Frankfurt am Main, Frankfurt, Germany; 4 Department of Anesthesiology, University Hospital Marburg, Marburg, Germany; 5 Department of Neurosurgery, University Hospital Giessen, Giessen, Germany; Georgia Regents University, UNITED STATES

## Abstract

**Background:**

Hypothermia has been discussed as playing a role in improving the early phase of systemic inflammation. However, information on the impact of hypothermia on the local inflammatory response is sparse. We therefore investigated the kinetics of local and systemic inflammation in the late posttraumatic phase after induction of hypothermia in an established porcine long-term model of combined trauma.

**Materials & Methods:**

Male pigs (35 ± 5kg) were mechanically ventilated and monitored over the study period of 48 h. Combined trauma included tibia fracture, lung contusion, liver laceration and pressure-controlled hemorrhagic shock (MAP < 30 ± 5 mmHg for 90 min). After resuscitation, hypothermia (33°C) was induced for a period of 12 h (HT-T group) with subsequent re-warming over a period of 10 h. The NT-T group was kept normothermic. Systemic and local (fracture hematoma) cytokine levels (IL-6, -8, -10) and alarmins (HMGB1, HSP70) were measured via ELISA.

**Results:**

Severe signs of shock as well as systemic and local increases of pro-inflammatory mediators were observed in both trauma groups. In general the local increase of pro- and anti-inflammatory mediator levels was significantly higher and prolonged compared to systemic concentrations. Induction of hypothermia resulted in a significantly prolonged elevation of both systemic and local HMGB1 levels at 48 h compared to the NT-T group. Correspondingly, local IL-6 levels demonstrated a significantly prolonged increase in the HT-T group at 48 h.

**Conclusion:**

A prolonged inflammatory response might reduce the well-described protective effects on organ and immune function observed in the early phase after hypothermia induction. Furthermore, local immune response also seems to be affected. Future studies should aim to investigate the use of therapeutic hypothermia at different degrees and duration of application.

## Introduction

Multiple injuries in trauma patients have been associated with severe systemic and local complications [1–4]. At the systemic level, a higher incidence of organ dysfunction has been reported. Locally, there is evidence for a higher incidence of delayed fracture healing and non-union in multiple trauma patients compared to those who sustain an isolated fracture [5–7]. These local and systemic complications have been associated with the posttraumatic immune response. Beside the well-known consequences of an overwhelming systemic inflammatory response on the development of remote organ damage, a profound local inflammation has also been assumed to negatively affect the early phase of bone repair thereby impairing fracture healing [6]. In this context, the immunologic milieu of local fracture hematoma in particular is supposed to be crucial for adequate initiation of repair processes (e.g. stimulation of angiogenesis, regulation of osteoclast activity) [8].

There is clear-cut evidence from experimental studies that induced hypothermia might exert beneficial effects on organ function in the early phase after hemorrhagic shock and severe tissue injury [9–12]. The clinical effects of induced hypothermia for the posttraumatic course are discussed controversially as the technique is applied to a very heterogeneous group of patients and in different clinical settings (i.e. after cardiac arrest, post-surgery) [13, 14]. As a potential pathomechanism, a beneficial modulation of the systemic pro-inflammatory immune response has been discussed. However, the long-term effects of induced hypothermia on systemic inflammation after re-warming remain unknown. Furthermore, the impact of a reduced body temperature on the local immunologic milieu of fracture hematoma has not been investigated specifically in the setting of combined trauma [15, 16].

We therefore aimed to investigate the long-term effects of induced hypothermia and re-warming on systemic and local inflammation. Additionally, the effects of induced hypothermia on the kinetics of local inflammation in fracture hematoma were analyzed. The study was performed in a clinically relevant, large animal model of combined trauma (chest and abdominal trauma, pressure-controlled hemorrhagic shock, tibia fracture).

## Material & Methods

### Animal care

All animal experiments have been performed in strict accordance with the guidelines of the Society of Laboratory Animal Science (GV-SOLAS) and the National Animal Welfare Law and after approval by the responsible government authority ("Regierungspräsidium Gießen/Hessen": Ref. MR 22/2013). The animals were handled according to guidelines of the Federation of European Laboratory Animal Science Association (FELASA). In total, 20 male pigs (*Sus scrofa*, 35 ± 5 kg, 3 months old) were used in this experiment. According to the above mentioned guidelines all experimental procedures (including catheterization, trauma, hemorrhage, resuscitation, ICU-treatment) were conducted under deep anesthesia and analgesia to minimize animal suffering and distress using propofol 3–4 mg kg/h and sufentanil 0.8 μg/kg/h during the entire study period of 48h. This setting was previously described by Eschbach et al. [17]. Consistent with the described guidelines and the EAST guidelines for resuscitation endpoints, endpoints were defined as earliest indicator in an animal of intractable pain, distress, suffering, or impending death during the study period [18]. Therefore animals were continuously (48h) monitored by an experienced physician and established criteria of deep anesthesia (loss of pedal reflex (toe pinch) and eye blink reflex) as well as markers of successful resuscitation, including restoration of normal blood pressure, heart rate, and urine output were recorded in accordance with the ATLS^®^ guidelines [19]. Compensated shock, described as ongoing metabolic acidosis [18], was monitored every 2h by blood gas analysis (pH, BE, lactate). Trauma related complications (i.e. tension pneumothorax) were treated according to the ATLS^®^ guidelines [19]. Under this close supervision, no unexpected deaths occurred during the entire study period. Euthanasia should be conducted in case resuscitation strategies were unsuccessful within 24h according to the EAST guidelines or if treatment of acute cardiopulmonary failure remained therapy-resistant (intractable medical condition: deranged pH (<7.40; >7.53, BE (<-15mmol/l), lactate (>5mmol/l) after 24h as any measured acid–base value exceeding these ranges was categorized as an indicator of prolonged acidosis) [18].

According to the described animal care guidelines, euthanasia was induced by enhancing anesthesia and applying an additional 100μg of sufentanil, 200mg of disoprivane, and 4 mg of pancuronium. Afterwards, 60–100mL of potassium chloride was administered until the occurrence of cardiac arrest.

### Group distribution

The Normothermia-Trauma group (NT-T, n = 15) included trauma animals that were kept normothermic over the entire observation period. In the Hypothermia-Trauma group (HT-T, n = 15) the body temperature was lowered to 33°C for 12 h. The sham groups were named Normothermia-sham (NT-S, n = 5) and Hypothermia-sham (HT-S, n = 5). As we acted in line with the accepted principal of the “3 Rs” (restriction, refinement, reduction), we compared the data of hypothermic animals in this study to results of a normothermic cohort gained from an identical experimental setting (NT-T: n = 15; sham: n = 5) [8, 17].

### General instrumentation and anesthesia

After a fasting period of 12 h, anesthesia was induced and pigs were intubated (7.5 mm tube). Ventilation was obtained in BiPAP mode using a defined volume of 6–8 ml/kg BW with a FiO_2_ of 30% and a PEEP of 5 mmHg, adjusted by capnometry targeting <7.5 kPa (Draeger, Evita, Danvers, MA, USA). Vital signs were monitored by electrocardiographic recording (ECG) and ECG-synchronized pulse oximetry. General anesthesia and analgesia was maintained by application of propofol and sufentanil during the entire study period. Fluids were additionally administered by continuous crystalloid infusion over the entire period (2 ml/kg/h). Single shot antibiosis was administered before the interventions (cefuroxime 1.5 g). An arterial pulse contour cardiac output (PiCCO, Pulsion Medical Systems, Germany) catheter (left femoral artery), a two lumen hemodialysis catheter (Arrow International, Germany) (left femoral vein), a central venous catheter (Arrow International, Germany) (right jugular vein) and a suprapubic catheter were inserted under sterile conditions. Operative tracheotomy was performed and a shortened 7.5 mm endotracheal tube was inserted.

### Induction of combined trauma and hemorrhage

Trauma consisted of a tibia fracture induced by placing the lower leg into a drop-weight device. A 20 kg plumb-cuboid was dropped from a height of 1.0 m. Afterwards a cast was applied. Blunt thoracic trauma was induced by applying a panel of 1 cm thickness to the right dorsal, lower chest. A bolt was shot onto this panel using cattle killing cartridges (9×17; Dynamit Nobel AG, Troisdorf, Germany) simulating blunt lung contusion. The shot was applied while the lungs of the animals were inflated, and inspiratory O_2_ was defined at 21% during the trauma period, simulating the ambient air. Next, a midline laparotomy was performed and the right upper liver lobe was explored. Using a sharp, custom-made, four-edged scalpel a penetrating abdominal injury was induced. After a short period of uncontrolled bleeding (approximately 30 s), liver packing was carried out with seven sterile packs of the same size. Laceration-associated bleeding was assessed macroscopically after 24 h, sterile packs were replaced and the abdomen was surgically closed. This evaluation and the documentation were performed by one experienced surgeon (DE). After hepatic packing, pressure-controlled and volume-limited hemorrhagic shock was induced by blood withdrawal, until a mean arterial pressure (MAP) of 30 ± 5 mmHg was reached. In this context, a maximum of 45% of total blood volume was drawn from the left femoral vein. The shed blood was abolished. Hemorrhagic shock was maintained for 90 min. Thereafter, the animals were resuscitated by adjusting baseline FiO2 (30%) and using crystalloid fluids in a volume of four times the shed blood volume over a period of 1 h. Then, hypothermia of 33°C was induced by the ARCTIC SUN^®^ 5000 temperature management system (Bard Medical). Pads with a hydrogel coating ensured contact with the animal’s skin. The conductive properties of the pads mimicked water immersion and animals were cooled down to 33°C within 3 h. This temperature was maintained for 12 h. Afterwards controlled re-warming (0.5°C per h) was performed and directed by the same temperature management device.

Sham animals and trauma animals were identically instrumented and received the same anesthetic and intensive care procedures. Respiratory and blood gas parameters were monitored and adjusted if necessary to keep parameters on a physiologic baseline level. Compared to trauma animals, sham animals were not subjected to any injury or hemorrhage but did receive therapeutic hypothermia as described above. In both groups (trauma animals and sham animals), blood samples were collected at the same time points.

### Data collection

Full blood samples were drawn immediately before induction of trauma (0 h), after trauma and resuscitation (2.5 h) and over the observation period at 14 h, 24 h and 48 h using Monovettes^®^ (SARSTEDT AG & Co, Germany). Fracture hematoma was extracted under sterile conditions by puncturing the fracture zone at 14 h, 24 h and 48 h and collected in an EDTA Monovette^®^ (SARSTEDT AG & Co, Germany). After centrifugation, serum was removed and stored at –80°C for further analysis. After an observation period of 48 h the animals were sacrificed. Data was collected using a Filemaker^®^ database (Filemaker Pro 5.0, Filemaker Inc.); access was limited by password protection. Security back up was done every 48 h.

### Laboratory evaluation

IL-6, IL-8, IL-10, HMGB1, and HSP70 were analyzed from serum samples using ELISA kits (IL-6, -IL-8, and IL-10: R&D systems, USA; HMGB1: IBL International GmbH, Germany; HSP70: USCN Life Science Inc., China) according to the manufacturer protocol. Fracture hematoma was analyzed using the same kits. All hematoma samples were centrifuged again at 4°C before usage. Referring to higher concentrations, all fracture hematoma samples were diluted (IL-6 1:10, IL-8: 1:4, IL-10: 1:4, HMGB1: 1:10, HSP70: 1:10).

### Statistics

Statistics were performed with SPSS (Version 22.0.0.0) using a Kolmogorow-Smirnow-Test for normal distribution, Student’s t-test for means (illustrated as mean ± SD), and Wilcoxon rank sum, Friedman and Kruskal-Wallis tests for statistical significance (p<0.05). Hemodynamic and physiologic parameters are presented as mean and standard deviation (illustrated as mean ± SD). Laboratory results are presented as mean and standard error of the mean (illustrated as mean ± SEM) Graphs were produced using Excel^®^ (Microsoft 2010).

## Results

### Survival

All animals in the HT-T group survived the observation period although cardiopulmonary resuscitation was necessary in 7% (n = 1). In contrast, normothermic animals showed a mortality of 13% (n = 2) in the NT-T group. Cause of death was cardiac arrest within the first 12 h of investigation (time of death: 1.48h and 6.05h after induction of trauma) due to ventricular fibrillation. Cardiopulmonary resuscitation was necessary in 26% (n = 4) of NT-T animals. Statistically significant differences between groups were not detected. All sham animals survived without the need for cardiopulmonary resuscitation.

### Hemodynamic and physiologic parameters

For shock induction, 45% of total blood volume was removed. MAP was 29 ± 7 mmHg in the NT-T group and 30 ± 8 mmHg in the HT-T group while maximal heart rate was 197 ± 28 beats/min in the NT-T group and 170 ± 40 beats/min in the HT-T group. No significant differences were observed between the HT-T and NT-T groups. When compared with the sham animals, a significant decrease of hemoglobin (Hb) levels was seen whereas lactate (Lac) levels significantly increased by the end of the induction phase in both trauma groups (Table 1). Base excess (BE) as well as bicarbonate (HCO_3_) significantly decreased in normothermic and hypothermic trauma animals when compared with the sham groups (Table 1). No significant changes were found between the HT-T and NT-T groups.

**Table 1:** Laboratory parameters.

	0min				90min			
Group	NT-T	NT-S	HT-T	HT-S	NT-T	NT-S	HT-T	HT-S
n	15	5	15	5	14	5	15	5
Hb	92 ±1.9	101 ±6.3	94 ±1.6	94 ±3.0	53 ±2.3	94 ±2.9	49 ±3.3	90 ±3.1
p-value	n.s.		n.s.		<0.001		<0.001	
BE	4.2 ±0.7	5.6 ±0.9	3.8 ±0.6	5.0 ±0.34	0.8 ±0.8	6.5 ±0.8	1.0 ±0.8	5.9 ±0.6
p-value	n.s.		n.s.		0.001		<0.001	
HCO^3^	29.10 ±0.36	29.48 ±0.74	28.49 ±1.03	29.16 ±0.27	25.97 ±0.63	30.32 ±0.85	23.84 ±1.82	30.28 ±0.56
p-value	n.s.		n.s.		0.002		<0.001	
LAC	0.89 ±0.10	1.26 ±0.21	1.15 ±0.70	0.82 ±0.14	3.01 ±0.41	1.02 ±0.12	2.98 ±0.34	0.78 ±0.08
p-value	n.s.		n.s.		0.001		<0.001	

*Systemic concentrations of inflammatory parameters (mean value* ± SEM): Hb (hemoglobin), BE (base excess), HCO3 (bicarbonate), Lac (lactate); n.s. (not significant)

### Systemic concentrations of inflammatory parameters

#### Cytokines

IL-6 concentrations at the baseline (0 h) did not reveal differences between the two trauma groups and the respective sham groups. At 14 h after trauma a significant increase of systemic IL-6 levels compared to baseline levels and respective sham animals was observed in both trauma groups (Table 2). No significant differences were found between the trauma groups at 14 h, 24 h and 48 h. However, systemic IL-6 levels tended to be higher in the HT-T group compared to normothermic animals. Correspondingly, normothermia also resulted in a more pronounced decrease of systemic IL-8 levels resulting in significantly lower concentrations compared to HT-T at 48 h after trauma (p = 0.004) (Table 2). Induction of hypothermia did not lead to significant differences of systemic IL-10 levels between the HT-T and NT-T groups over the entire study period.

**Table 2:** Serum cytokine & alarmin concentrations (mean value ± SEM); NT-T (normothermic trauma), NT-S (normothermic sham), HT-T (hypothermic trauma), HT-S (hypothermic sham), statistical significance p = <0.05 (^a)^ from sham, ^b)^ from baseline, ^c)^ NT-T vs. HT-T).

		0h	2.5h	14h	24h	48h
**IL-6 (pg/ml)**	NT-T	32.29 ±24.99	41.10 ±25.60	62. 35 ±38.12 ^a) b)^	71.95 ±54.76 ^a) b)^	79.69 ±58.59 ^a) b)^
	NT-S	29.80 ±35.22	24.04 ±17.64	22.48 ±14.26	24.07 ±14.63	30.87 ±23.28
	HT-T-S	42.28 ±14.66	41.54 ±24.73	82.01 ±47.48 ^a) b)^	86.23 ±35.93 ^a) b)^	114.30 ±70.74 ^b)^
	HT-S	40.44 ±2.67	52.29 ±5.41^b)^	41.71 ±20.31	43.22 ±16.75	66.29 ±35.37
**IL-8 (pg/ml)**	NT-T	156.29 ±32.96	140.62 ±41.24	148.90 ±25.85	161.78 ±32.92 ^a)^	139.71 ±34.44
	NT-S	161.72 ±13.50	161.58 ±9.96	178.99 ±5.01	108.90 ±36.92	166.29 ±43.33
	HT-T	168.53 ±38.97	163.46 ±19.16 ^a)^	157.41 ±24.16 ^a)^	174.79 ±24.97 ^a)^	181.31 ±42.00 ^a) c)^
	HT-S	181.48 ±10.43	227.09 ±27.17	311.96 ±193.20	259.43 ±32.53	230.07 ±24.88
**IL-10 (pg/ml)**	NT-T	57.94 ±37.82	65.35 ±42.25	64.56 ±41.83	64.55 ±37.31	59.50 ±30.24
	NT-S	76.54 ±38.79	82.69 ±47.93	77.45 ±45.21	76.22 ±32.22	78.58 ±41.04
	HT-T	37.99 ±38.90	46.19 ±25.87	50.41 ±33.99 ^a)^	69.53 ±61.40 ^b)^	72.37 ±69.29 ^b)^
	HT-S	38.50 ±4.50 ^c)^	70.28 ±15.6 ^b)^	82.52 ±24.33 ^b)^	98.65 ±38.27 ^b)^	106.38 ±50.08 ^b)^
**HMGB1 (ng/ml)**	NT-T	1.25 ±0.87	2.53 ±1.58 ^a) b)^	2.20 ±1.54	4.80 ±3.94 ^a) b)^	3.01 ±3.21
	NT-S	0.54 ±0.61	0.45 ±0.26	1.40 ±0.65	1.08 ±0.53	1.77 ±0.35
	HT-T	1.54 ±1.23	3.83 ±1.19 ^a) b)^	3.70 ±2.56	4.81 ±2.49 ^a) b)^	4.73 ±2.30 ^a) b) c)^
	HT-S	1.78 ±0.88	1.73 ±0.49	1.63 ±1.33	1.22 ±0.91	1.46 ±1.17
**HSP70 (ng/ml)**	NT-T	87.75 ±59.08	54.21 ±45.71^a) b)^	47.04 ±40.07 ^a) b)^	56.06 ±42.95 ^b)^	75.96 ±59.36
	NT-S	129.92 ±47.00	131.04 ±33.52	110.19 ±34.71	83.89 ±19.63	79.14 ±36.73
	HT-T	50.74 ±39.73	31.95 ±37.48 ^b)^	18.98 ±21.05 ^b) c)^	16.03 ±14.53 ^b) c)^	11.51 ±9.79 ^b) c)^
	HT-S	53.37 ±32.71	54.95 ±40.98	49.92 ±39.50	42.27 ±39.79	32.63 ±34.96 ^b)^

#### Alarmins

At 2.5 h, trauma resulted in significantly increased HMBG1 concentrations when compared to both baseline values and respective sham animals (Table 2). Systemic HMGB1 levels demonstrated a prolonged increase in the HT-T group (Table 2) resulting in significantly higher concentrations in the HT-T group at 48 h.

Both trauma groups showed a decrease of anti-inflammatory HSP70 after trauma at 2.5 h, compared to the baseline and the respective sham group at 2.5 h (Table 2). NT-T animals demonstrated a recovery towards baseline values at 48 h, whereas a progressive decrease of serum concentrations was observed in the HT-T group. This resulted in significant differences compared to the NT-T group at 14 h (p = 0.033), 24 h (p = 0.002) and 48 h (p<0.001) (Table 2).

### Local concentrations of inflammatory parameters

#### Cytokines

**Fig 1:**
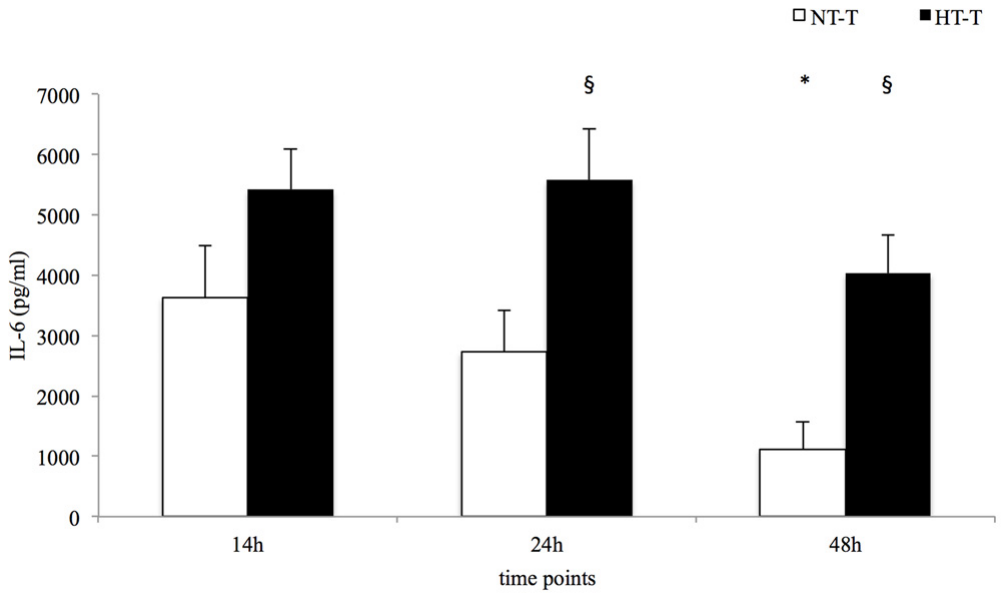
Local IL-6 concentrations. Statistical significance p = <0.05 (* from baseline, ^§^ NT-T vs. HT-T).

**Fig 2:**
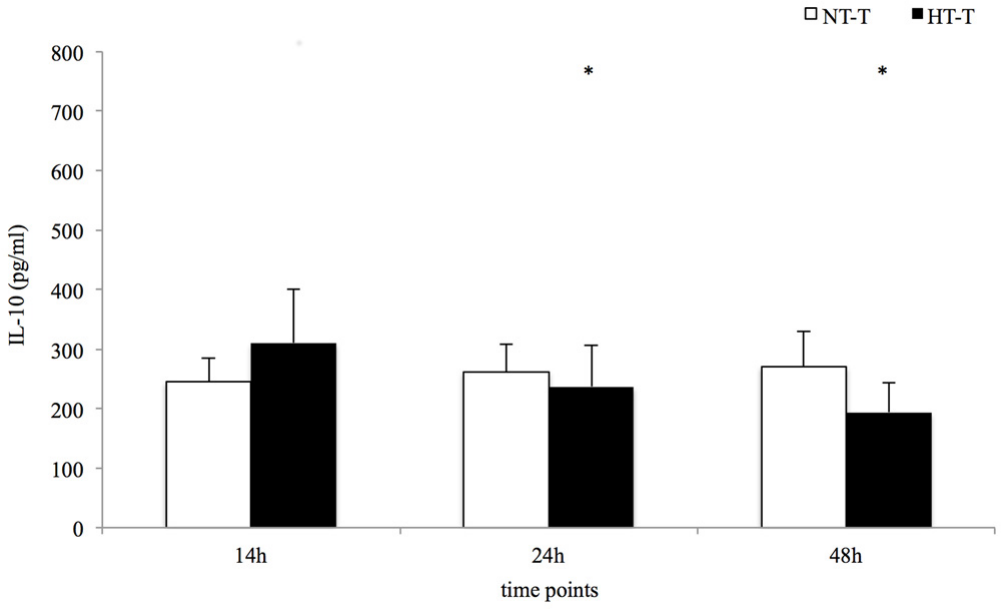
Local IL-10 concentrations. Statistical significance p = <0.05 (* from baseline).

Local levels of IL-6, IL-8 and IL-10 were significantly higher in the fracture hematoma compared to systemic serum levels in both trauma groups at 14 h, 24 h and 48 h after trauma induction (Table 3). The HT-T group demonstrated a more prolonged increase of IL-6 compared to the NT-T group resulting in significantly higher levels at 24 h and 48 h (Table 3 and Fig 1). IL-8 concentrations showed an increase over time in both the NT-T and HT-T groups (Table 3). In normothermic trauma animals, no significant changes of local IL-10 concentrations were observed, whereas the HT-T group presented a significant decrease over time (Table 3 and Fig 2).

**Table 3:** Serum & fracture hematoma concentrations.

		14h		24h		48h	
		NT	HT	NT	HT	NT	HT
**IL-6 (pg/ml)**	serum	62. 35 ±38.12	82.01 ±47.48	71.95 ±54.76	86.23 ±35.93	79.69 ±58.59	114.30 ±70.74
	hematoma	3631.65 ±2992.31^a)^	5498.08 ±2497.62^a)^	2725.91 ±2370.57^a)^	5456.53 ±3091.76^a)^ ^c)^	1120.99 ±1561.31^a)^ ^b)^	4142.74 ±2377.71^a)^ ^c)^
**IL-8 (pg/ml)**	serum	148.90 ±25.85	157.07 ±25.11	161.78 ±32.92	174.79 ±24.97	139.71 ±34.44	181.31 ±42.00
	hematoma	658.29 ±84.11^a)^	370.37 ±94.68 ^a)^ ^c)^	676.30 ±181.66 ^a)^	379.12 ±82.11 ^a)^ ^c)^	877.69 ±491.47^a)^ ^b)^	394.07 ±115.71 ^a)^ ^c)^
**IL-10 (pg/ml)**	serum	64.56 ±41.83	50.41 ±33.99	64.55 ±37.31	69.53 ±61.40	59.50 ±30.24	72.37 ±69.29
	hematoma	245.57 ±131.97^a)^	345.10 ±344.49 ^a)^	262.10 ±163.15 ^a)^	264.57 ±269.27 ^a)^ ^b)^	271.47 ±193.26 ^a)^	219.21 ±178.53^a)^ ^b)^
**HMGB1(ng/ml)**	serum	2.20 ±1.54	3.70 ±2.56	4.80 ±3.94	4.81 ±2.49	3.01 ±3.21	4.73 ±2.30
	hematoma	573.50 ±504.35^a)^	593.75 ±303.66 ^a)^	351.89 ±152.79 ^a)^	594.62 ±158.86 ^a)^ ^c)^	246.79 ±135.00 ^a)^ ^b)^	504.95 ±212.52 ^a)^ ^c)^
**HSP70 (ng/ml)**	serum	47.04 ±40.07	18.98 ±21.05	56.06 ±42.95	16.03 ±14.53	75.96 ±59.36	11.51 ±9.79
	hematoma	87.17 ±95.82	47.01 ±32.88^a)^	84.75 ±99.81	34.18 ±21.73^a)^	130.61 ±256.02	38.51 ±28.88^a)^

(mean value ± SEM); NT (normothermic), HT (hypothermic); statistical significance p = <0.05

^a)^ from systemic concentrations,

^b)^ from baseline,

^c)^ NT-T vs. HT-T

#### Alarmins

**Fig 3:**
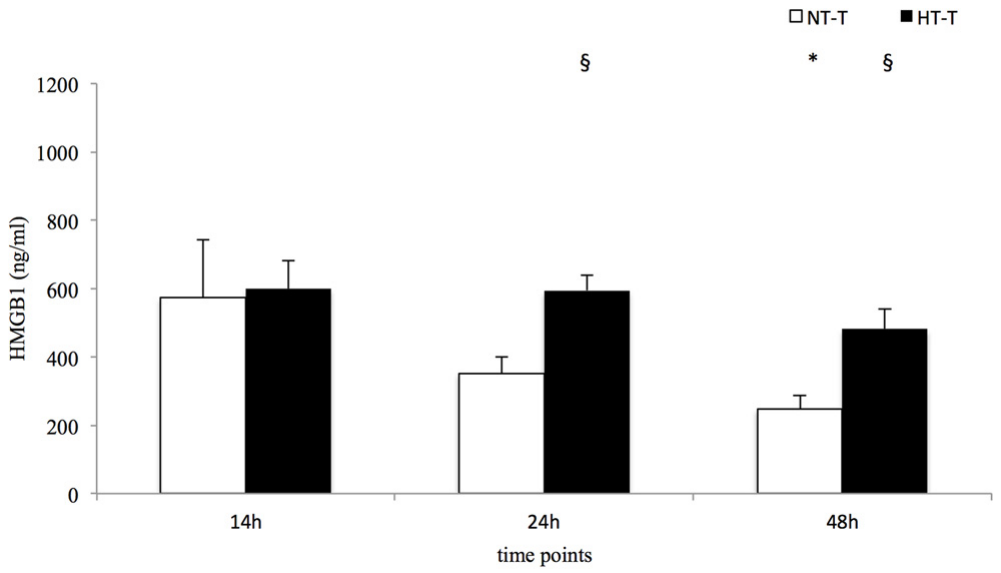
Local HMBG1 concentrations. Statistical significance p = <0.05 (* from baseline, ^§^ NT-T vs. HT-T).

Local levels of pro-inflammatory HMGB1 were higher compared to systemic concentrations in both trauma groups (Table 3). A significant decrease was found in normothermic pigs, while concentrations in hypothermic animals remained stable until 24 h, resulting in significant differences between the groups at 24 h (p = 0.002) and 48 h (p = 0.002) (Table 3 and Fig 3). In contrast, local HSP70 levels in the NT-T group only tended to be higher when compared to the systemic serum concentrations, while local HSP70 concentrations in the HT-T group were significantly higher at every time point (Table 3).

## Discussion

The systemic as well as the local immunologic response seem to have a crucial impact on the development of posttraumatic complications [6, 16, 20]. Although beneficial effects of induced hypothermia on early systemic inflammation and organ function were demonstrated in previous experimental studies, the long-term effects of a therapeutic reduction of body temperature and subsequent re-warming have not been investigated. Furthermore, the impact on the local immunologic milieu in fracture hematoma has not yet been investigated [12, 21–23]. These aspects limit the potential clinical applicability for the treatment of multiple trauma patients. Therefore, we aimed to investigate the long-term effects of induced hypothermia on local and systemic inflammation in a clinically relevant porcine model of combined trauma.

The main results of our study might be summarized as follows:

1. In the late posttraumatic phase (>14 h after trauma), induced hypothermia resulted in a prolonged pro-inflammatory systemic response when compared to normothermia.
2. In local fracture hematoma, concentrations of both pro- and anti-inflammatory mediators were markedly increased when compared to systemic concentrations.
3. Hypothermia was associated with prolonged elevated levels of local pro-inflammatory mediators while local anti-inflammatory mediators continuously decreased.

### Therapeutic hypothermia and systemic immune response

Induced hypothermia has been described as reducing the early inflammatory response after trauma. For the later posttraumatic course, however, the impact of hypothermia remains less clear, as unchanged or even increased levels after re-warming have been assumed [24–27]. In the current study we observed a prolonged increase of HMGB1 after induction of hypothermia and re-warming. These increased concentrations after re-warming have also been described for other clinical conditions, such as cardiac arrest [26]. An i*n vitro* study also found evidence for an increased HMGB1 release of endothelial cells under hypothermic conditions [28]. This increase was associated with hypothermia-induced tissue necrosis. As HMGB1 is known to stimulate IL-6 production, this might also explain the prolonged increase of systemic IL-6 seen in this and other studies [29, 30]. Song et al. concluded that prevention of both accidental and induced hypothermia might reduce the release of pro-inflammatory mediators and the associated immunologic imbalance with its potential clinical complications (e.g. remote organ damage) [28, 31]. However, beside its pro-inflammatory characteristics different studies also reported about significant anti-inflammatory properties of IL-6 with a complex biology as well as a specific trans-signaling mechanisms after infectious and traumatic results [32]. Furthermore, IL-6 has been proven to play a central role in the setting of infections, as it is required for resistance against different bacteria [32].

The findings of our study have to be considered for the potential use of induced hypothermia in the clinical setting, as the impact on inflammation seems to be effective only for a short time period after re-warming. Furthermore, in accordance with previous studies, we also observed the well-described anti-inflammatory effects of induced hypothermia for the late posttraumatic phase [10, 27, 33]. These results support the hypothesis that the anti-inflammatory effects of hypothermia and the associated risk for infectious complications persist for a prolonged period after re-warming.

In conclusion, our results indicate that induced hypothermia and re-warming might result in a prolonged immunologic imbalance compared to normothermic controls, which might result in systemic complications [34–36]. Therefore, the long-term effects of induced hypothermia in particular have to be investigated in further experimental studies, before considering hypothermia for the treatment of multiple trauma patients.

### Therapeutic hypothermia and immune response in early fracture hematoma

Previous studies found evidence that cytokines (e.g. IL-6) are of major importance in the early phase of fracture healing [37, 38]. In accordance with results from small animal models, we also observed locally increased IL-6 levels in fracture hematoma in the acute phase after combined trauma [8, 39, 40], with decreasing IL-6 values over the further clinical course [8, 41]. Induced hypothermia, however, resulted in a prolonged increase of local IL-6. As the effects of IL-6 on early fracture healing seem to be concentration-dependent, this might have significant clinical implications [16, 42]. In this context De Benedetti et al. demonstrated that overexpression of IL-6 resulted in severe osteopenia with reduced osteoblast and increased osteoclast numbers and activity [42]. Accordingly, Recknagel et al. demonstrated in a rat model that additional chest trauma resulted in a local increase of IL-6 concentration at the fracture site that was associated with impaired bone healing [41].

Additionally, pro-inflammatory HMGB1 has also been proven to play a crucial role in tissue repair and bone regeneration after trauma [43]. Despite this potentially relevant role of HMGB1, information on local concentrations at the fracture site is not available in the literature. In our combined trauma model, a significant increase of HMGB1 was observed. Comparable to local IL-6 levels, induced hypothermia resulted in a prolonged increase of HMGB1 at the fracture site. Accordingly, Morita et al. found locally increased HMGB1 levels in bronchoalveolar lavage fluid after induction of hypothermia compared to normothermic conditions. This confirms that body temperature seems to have an impact on local HMGB1 expression [44]. As HMGB1 has been described to induce IL-6 synthesis, increased HMGB1 values might be responsible for high local concentrations of this secondary cytokine in the fracture hematoma [45, 46]. Whether the hypothermia-induced prolongation of increased pro-inflammatory mediator levels in early fracture hematoma has a negative effect on the onset of bone healing has to be addressed in further studies [16].

In accordance with an isolated fracture hematoma model [20], we also found an increase of local IL-8 concentrations in normothermic animals. However, induced hypothermia resulted in an attenuated increase of IL-8 levels. Other studies in small [47] and large [48, 49] animals found comparable results for other body compartments. In this context, Fröhlich et al. described a five-fold reduction of hepatic IL-8 expression after induction of hypothermia in a porcine trauma model [12]. These different effects of induced hypothermia on local IL-6 and IL-8 levels might be explained by the kinetics of migration of immune competent cells into the fracture hematoma. In this context, IL-6-producing T-cells and neutrophils have been shown to migrate much faster into the fracture hematoma representing about 87% of all specified cells in the fracture hematoma 1 and 4 h after trauma (compared to 7% monocytes) [50]. Therefore, these cells might already have entered the hematoma before induction of hypothermia. In contrast, migration of monocytes in the later phase might be affected by hypothermia (e.g. due to a reduction of blood flow, decreased monocyte activity). As monocytes have been identified as a main source of IL-8 [51], this might result in a reduction of IL-8 synthesis [23].

The prolonged local pro-inflammatory milieu observed in hypothermic trauma animals also seems to affect the anti-inflammatory response at the fracture site. In this context, a pro-inflammatory environment has been shown to reduce monocyte differentiation towards IL-10-producing macrophages (phenotype M2) [52–55]. The extended pro-inflammatory response after hypothermia induction might therefore also explain the reduced local IL-10 concentrations in our study. As IL-10 represents a potential key component in the initiation of the regenerative healing process [55, 56], a prolonged pro-inflammatory milieu with reduced IL-10 levels might impair bone healing. In this context, IL-10 has been demonstrated to influence bone resorption [57, 58] and to enhance bone healing [59]. A deficit of IL-10 results in osteopenia, mechanical fragility of bones and defects in their formation [60]. We also observed decreased concentrations of anti-inflammatory HSP70. However, data regarding the observed decrease of this alarmin have to been interpreted with caution, as HSP70 release is normally induced by an increase of body temperature (i.e. pyrexia in sepsis) and might by suppressed due to the cold exposure via induction of hypothermia.

### Therapeutic hypothermia and survival

Induction of therapeutic hypothermia resulted in improved outcome after ischemia and reperfusion in patients with cardiac arrest and cerebral injuries [61, 62]. In contrast to these entities, multiple trauma patients frequently suffer from hypovolaemic shock with its potential consequences, such as the lethal triad of death (accidental hypothermia, acidosis and coagulopathy) [63]. In this context, a close association of accidental hypothermia and adverse outcome after severe trauma has been demonstrated [22, 64]. Although active rewarming of patients with accidental hypothermia is currently the only available method to improve patient outcome, experimental data has given evidence that induction of therapeutic hypothermia after hemorrhage control might exert beneficial effects [12, 63, 65, 66]. In this context, George et al. found a significantly lower mortality rate in animals subjected to mild and moderate hypothermia after trauma, which is in accordance to the trend in our study. However some relevant differences between our study and the experiments of George et al. have to be considered. Firstly, the trauma severity induced in the study of George et al. induced was less severe as in our study. Secondly, the authors used ice bags instead of a temperature-controlled device. Lastly, the duration of hypothermia was shorter in the study of George et al. [65]. This might explain the early death of the two animals within our group [65]. Still, we observed comparable causes of death (circulatory collapse) compared to George et al. Yet, literature regarding induced hypothermia is increasing and future studies might reveal new insights of the method itself as well as a suitable patient population.

### Strengths and limitations

As systemic as well as local inflammation has an impact on the development of organ dysfunction and bone regeneration, we aimed to elucidate early kinetics of both the systemic and local inflammatory responses in a clinically relevant, large animal model of combined trauma subjected to therapeutic hypothermia. This model is unique as it has a posttraumatic observation time of 48 h under intensive care conditions (with mechanical ventilation and intensive care treatment) including a hypothermia period of 12 h. Although important information can be drawn from the study, our data do not permit conclusions on the cellular mechanisms that regulate local or systemic inflammatory response nor can any assumptions on the interaction with osteo- and chondrogenesis be made. Furthermore our results affect a very specific patient population and can possibly not be transferred one to one to other patient populations (i.e. cardiac arrest, post-surgery). The above mentioned limitations and problems will be the focus of an ongoing follow-up study.

### Conclusion

In light of the current results with prolonged systemic inflammation as well as a significant modulation of the local inflammatory response, a significant impact of induced hypothermia on the posttraumatic course has to be assumed. How far these changes affect the incidence of systemic (e.g. organ dysfunction, infections) and local (e.g. bone healing) complications has to be investigated in further studies. Knowledge about the relevance of different aspects, such as cooling rate, depth and length of hypothermia as well as re-warming period are needed before implementation of induced hypothermia for the treatment of multiple trauma patients can be considered.
